# Feasibility of Ethos adaptive treatments of lung tumors and associated quality assurance

**DOI:** 10.1002/acm2.14311

**Published:** 2024-02-22

**Authors:** Sonja Wegener, Stefan Weick, Robert Schindhelm, Jörg Tamihardja, Otto A. Sauer, Gary Razinskas

**Affiliations:** ^1^ Department of Radiotherapy and Radiation Oncology University of Wurzburg Wurzburg Germany

**Keywords:** adaptive radiotherapy, EPID dosimetry, lung, secondary dose calculation, synthetic CT

## Abstract

**Motivation:**

Online adaptive radiotherapy with Ethos is based on the anatomy determined from daily cone beam computed tomography (CBCT) images. Dose optimization and computation are performed on the density map of a synthetic CT (sCT), a deformable registration of the initial planning CT (pCT) onto the current CBCT. Large density changes as present in the lung region are challenging the system.

**Methods:**

Treatment plans for Ethos were created and delivered for 1, 2, and 3 cm diameter lung lesions in an anthropomorphic phantom, combining different insets in the pCT and during adaptive and non‐adaptive treatment sessions. Primary and secondary dose calculations as well as back‐projected dose from portal images were evaluated.

**Results:**

Density changes due to changed insets were not considered in the sCTs. This resulted in errors in the dose; for example, ‐15.9% of the mean dose for a plan when changing from a 3 cm inset in the pCT to 1 cm at the time of treatment. Secondary dose calculation is based on the sCT and could therefore not reveal these dose errors. However, dose calculation on the CBCT, either as a recalculation in the treatment planning system or as pre‐treatment quality assurance (QA) before the treatment, indicated the differences. EPID in‐vivo QA also reported discrepancies between calculated and delivered dose distributions.

**Conclusions:**

An incorrect density distribution in the sCT has an impact on the dose calculation accuracy in the adaptive treatment workflow with the Ethos system. Additional quality checks of the sCT can detect such errors.

## 1 INTRODUCTION

Radiotherapy treatment plans are usually based on the patient's anatomy at the time of the initial computed tomography (CT) used for planning. Both, inter‐ and intra‐fractional anatomical changes may occur during the course of treatment. Adaption of the treatment plan for lung cancer patients allows a reduction of the planning target volume (PTV) and consequently a reduction of dose to the surrounding tissue.^1^, ^2^, ^3^ Anatomical changes such as continuous shrinkage^1^ or atelectasis^2^ are typical indications for offline adaptive radiotherapy. Online adaptive radiotherapy can be performed in different ways, either with a plan library approach^4^ or with adaption of the plan to the anatomy of the day as seen in magnetic resonance^5^ or cone beam CT (CBCT) imaging.^6^ The Ethos system allows CT‐based image‐guided online adaptive radiotherapy.^7^ The successful implementation and treatment data have mainly been reported for the pelvic region.^6^, ^8^, ^9^ Other regions, such as head and neck^10^ or lung^11^ treatments have been studied in silico using an emulator.

The workflow for the adaptive treatment on Ethos requires the acquisition of a planning CT (pCT) and the generation of an initial reference plan. During each session, a CBCT is generated and a synthetic CT (sCT) is created by deformable image registration of the pCT to the CBCT.^12^ Daily dose calculation is performed on the sCT.^7^ Dose calculation directly on CBCT images is typically avoided due to challenges with the Hounsfield unit to density conversion and increased motion artifacts.^13^, ^14^, ^15^ Additionally, the system detects organ structures on the CBCT called “influencers,” which are reviewed by the users and modified when necessary. The influencer structures are taken into account when propagating target structures from the pCT onto the CBCT image.^7^ Those are also reviewed and may be modified, before generating two treatment alternatives, that is, the recalculation of the reference plan on the current sCT called “scheduled plan” and a newly optimized plan with the underlying sCT density referred to as “adapted plan.” Therefore, the correctness of the dose calculation depends on the correct densities in the sCT. Currently, there is no quality assurance (QA) of the sCT implemented in Ethos.

Studies on the feasibility of adaptive treatments with Ethos for anatomical regions typically only focus on the quality of automatic contouring and plan quality.^10^, ^11^ Studies include body regions with large density gradients and expected anatomical density changes, but the role of the sCT was not specifically addressed. In a phantom study mimicking anatomical changes, gas present during the pCT but not during the CBCT was identified as an issue: Point doses differed by around 3% between calculation during the adaptive session and measurement when the density was not overridden.^16^

Here, the dose calculation accuracy in a body region of heterogeneous density was investigated. The feasibility to detect such calculation errors with available means of quality assurance (QA) was evaluated. To this end, a lung phantom with variable tumor insets was irradiated following the Ethos adaptive workflow choosing either the scheduled or adapted plan. The generated sCTs, the dosimetric effect of calculating on sCTs compared to calculating on the actual geometry, and the results of plan‐specific QA are evaluated in order to understand both the limitations of the system and to identify suitable QA methods.

## 2 METHODS

### 2.1 Planning CT and treatment plan

**FIGURE 1:**
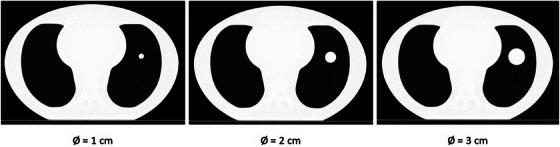
pCTs of the anthropomorphic lung phantom with the three different insets.

Planning CTs were acquired of a CIRS Dynamic Thorax Phantom (Sun Nuclear, Melbourne, FL, USA) with a SOMATOM go.Open Pro scanner (Siemens Healthineers, Erlangen, Germany) using the thoracic preset (120 kV). Rods with spheres of three different diameters were inserted into the phantom mimicking solid lung lesions of different sizes (Figure 1). CTs were acquired with each of the nominal 1, 2, and 3 cm diameter spheres inserted. Actual diameters measured in the pCT were approximately 0.8, 1.8, and 2.8 cm. The phantom allows for longitudinal and rotational movement of the rods including the spheres, but they were kept static throughout this study.

All three planning CTs were reconstructed with a slice thickness of 2 mm and imported as three separate patients into the radiotherapy (RT) Intent “Lung Left” in Ethos Treatment Management software (version 2.1, Varian Medical Systems, Palo Alto, CA, USA). The solid sphere was contoured as the clinical target volume (CTV). A planning target volume (PTV) was derived from the CTV by isotropically adding a 5 mm margin. Additionally, both lungs, defined as influencers, the heart, and the body contours were created.

For each planning CT, a treatment plan with nine equidistant IMRT fields was generated aiming for a CTV mean dose of 2 Gy per fraction for a total of 50 fractions. The large number of fractions allowed for multiple irradiations of the same plan in the clinical mode. The complete list of clinical goals for plan optimization is found in Table 1. The calculation in the Ethos planning system was performed with the Ethos Acuros XB beam model. The calculation grid size was set to 2.5 mm, which is the smallest grid size available.

**TABLE 1:** List of clinical goals for plan optimization.

Target Volume/Organ	Target	Priority
CTV	Dmean ≥ 99%	1
	Dmean < 101%	1
PTV	D99% > 96%	1
	D1% < 103%	2
	D50% > 99.5%	2
	D50% < 100.5%	2
Lung Left	Dmean ≤ 12 Gy	2
	D10% ≤ 20 Gy	3
Lung Right	Dmean ≤ 4 Gy	4
	D10% ≤ 6 Gy	3
Heart	D1% ≤ 10 Gy	4

### 2.2 On‐couch sessions

The treatment sessions were carried out using an Ethos linear accelerator (Halcyon version 3.1, Varian Medical Systems). The phantom including an insert was placed centrally on the couch. CBCT was acquired using the thoracic preset (125 kV, 294 mAs, 49.2 cm diameter). For each combination of insets present during the pCT and treatment, two irradiations were performed: one using the scheduled plan, one using the adapted plan. To study reproducibility, for the combination of 3 cm diameter sphere in the pCT and 2 cm diameter sphere during treatment three irradiations for each scheduled and adapted plan were carried out.

In detail, the CBCT and the influencers were reviewed and accepted without any changes to the structures. The review is a necessary step to proceed in the workflow although the influencer structures were reliably and reproducibly identified due to the static nature of the phantom. The CTV was manually adapted to the sphere when the proposed target structure did not fit visually. Either the scheduled plan or the adapted plan was irradiated. Just for technical reasons, after irradiation, another CBCT in the treatment position was performed, in order to be able to export the complete session data. All session data was then exported from the Ethos Treatment Management software for further use of the treatment plan, dose distribution, CBCT and sCT data, and the portal images.

### 2.3 Plan parameter evaluation

During treatment sessions, data are regularly exported to the software Mobius3D (version 4.0.2, Varian Medical Systems) for the purpose of a secondary dose check. While the user still reviews the target structures during the adaptive session, an adapted plan is created in the background. This plan and the scheduled plan are exported and calculated in Mobius3D. This first export includes the structure set of the reviewed influencer structures and the automatically contoured targets. Therefore, the volume of the CTV before editing was obtained from the generated Mobius3D calculation report. After editing the target structures and creation of a new adaptive plan, another automatic export takes place, so the CTV volume after editing was obtained from the calculation report of this subsequent export. The monitor units (MU) of all irradiated adapted plans were also obtained from the Mobius3D calculation report.

### 2.4 Evaluation of sCT

The pCTs and the sCTs of the adaptive session were read into MatLab (version R2020a, MathWorks, Natick, MA, USA). Both datasets cover the same volume of interest and have identical data size. The Hounsfield Units (HU) were converted into mass density using the same calibration curve as in the treatment planning system (TPS). A line profile through the center of the inserted sphere was extracted for each dataset to compare the density and assess the size of the sphere as presented in the different CT images.

### 2.5 Recalculation in eclipse

Ethos session data was imported into Eclipse (version 15.6, Varian Medical Systems, Palo Alto, CA, USA) and dose distributions were calculated for the following settings:

- pCT with the initial plan created in Ethos Treatment Management,
- sCT with the irradiated plan (either scheduled or adaptive),
- CBCT with the irradiated plan (either scheduled or adaptive),
- pCT with the irradiated plan (either scheduled or adaptive).

Care was taken to maintain the correct position of the isocenter used during the actual treatment in the calculation. Dose volume histogram (DVH) parameters were extracted.

For pCT and sCT, the Hounsfield to density assignment as already established in the TPS and identical to the one used in the Ethos Treatment Management software was used. For the Ethos CBCT, calibration was acquired using the Advanced Electron Density Phantom (Sun Nuclear Corporation, Melbourne, FL, USA) including all inserts except the metallic ones.

### 2.6 Secondary dose calculation

Secondary dose calculation was performed in Mobius3D and in RadCalc (version 7.2.2, LifeLine Software Inc., Tyler, TX, USA) both, on the sCT and on the CBCT. Mobius3D as the standard tool for plan verification with Ethos was pre‐commissioned by the vendor. Only the dynamic leaf guide correction factor was adjusted according to the manufacturer's procedure and set to 0.40. The calculation on the sCT was carried out automatically parallel to the treatment sessions. To calculate on the CBCT, the Hounsfield calibration data in the software and the scanner name in the DICOM file had to be changed before manually placing the data into the Mobius3D import folder.

RadCalc also provided a pre‐configured beam model for Ethos or Halcyon, which was used. pCT and CBCT data were imported as separate patients using the respective Hounsfield calibrations. The plans were recalculated using the Collapsed Cone algorithm, fine dose grid spacing (0.2 cm grid size), DVH sampling size of 0.2 cm, and bin size of 1 cGy.

Gamma analysis in Mobius3D was carried out using the 3%/2 mm criterion, absolute dose, 10% low dose threshold for the whole geometry, or restricted to the PTV. DVH parameters were extracted.

In RadCalc, gamma analysis was performed using the same criterion. DVH parameters were obtained.

### 2.7 EPID‐dosimetry

During all treatments, portal images were recorded on the electronic portal imaging detector (EPID) of the Ethos treatment machine and afterwards processed in RadCalc.

The commissioning of the EPID model in RadCalc had been performed by the manufacturer based on images of specified fields with different sizes and various amount of water‐equivalent material between the radiation source and the imager as per their specifications.

In the RadCalc software, EPID images were added to the respective treatment plan, and the software calculated dose and DVH information based on a back projection algorithm. Gamma analysis and DVH parameters were obtained from the calculations for both a projection back into the sCT and into the CBCT.

## 3 RESULTS

### 3.1 Adaption process

**FIGURE 2:**
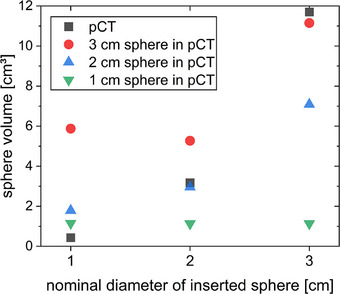
Volume of the automatically generated CTV contours during the adaption process. The CTV coincides with the volume of the inserted sphere in the pCT used for planning. Volumes for different combinations of inserted spheres during the treatment CBCT (x‐axis) and the pCT (distinguishable by different symbols) are displayed. The volume of the sphere in the pCT is indicated (open squares) as a reference.

The volume of the automatically generated contour of the CTV matched the expected volume of the sphere when the same inset was present during pCT and CBCT, but deviated when the inset was changed between the two images (Figure 2). For the latter cases, the contoured CTV only partly adapted to the new size when the 2 and 3 cm diameter spheres were used in the pCT. For the 1 cm sphere in the pCT, the automatically contoured CTV remained almost constant. Therefore, manual adjustment of the CTV contours was required in all cases where a combination of different insets during CBCT and pCT was used. The maximum deviation of 10.6 cm^3^ in volume occurred when having a 1 cm diameter inset in the pCT and a 3 cm diameter inset during treatment. This means that the volume of the inset obtained from the sCT is only around 10% of the volume of the inset actually inserted at that time. When having a 3 cm diameter inset in the pCT and a 1 cm inset during treatment, the volume is 5.4 cm^3^ larger in the sCT. This means that the volume of the inset obtained from the sCT is more than 10‐fold the volume of the inset actually inserted.

**FIGURE 3:**
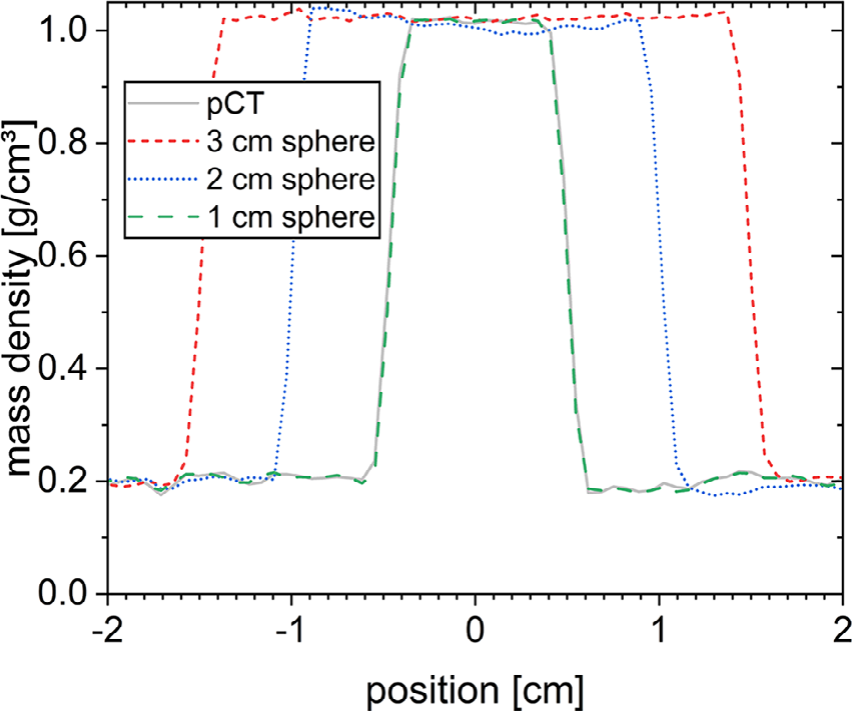
Profiles through the density distribution of the sCTs generated during adaptive treatment. Different spheres (indicated in the legend) were present at the time of the pCT, the 1 cm sphere was inserted during all CBCT acquisitions.

The density profile centrally through the sphere was almost identical in the sCT and the pCT when the same inset was present in both CTs (Figure 3). The density distribution in the sCT in the sphere region did not adapt to the size of the inserted spheres when insets were changed between the pCT and the CBCT. Even though a different inset was present in the CBCT, the diameter of the higher‐density region remained as in the pCT. The observations were the same for all nine studied combinations of insets during pCT and CBCT. Thus, only the example of the 1 cm sphere inserted during the treatment session is shown in Figure 3. Summarized, neither the auto‐contouring of the target volume structures nor the density distributions of the sCT are correctly assigned when the high‐density target changes volume between the initial pCT and treatment.

**FIGURE 4:**
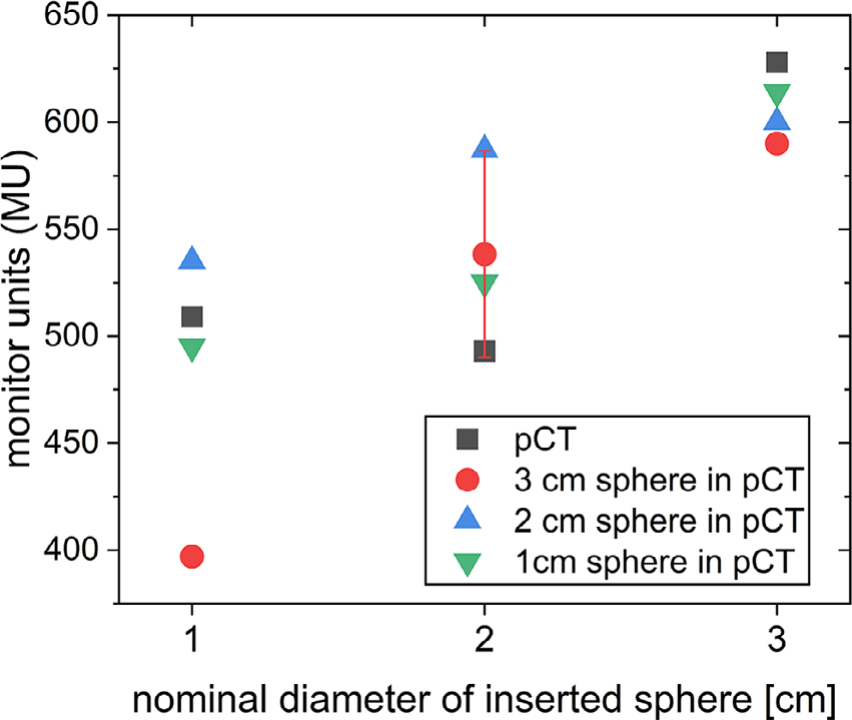
Number of MU of the adapted plans as a function of the inserted sphere diameter for different insets present in the pCT (different symbols as indicated in the legend). The number of MU for the initial plan on the pCT is given as a reference.

The MU of the adaptive plans generated with Ethos based on the sCT and the reviewed CTV contours are shown in Figure 4. Neither do the adapted plans always show a comparable number of MU to the initial plan for the unchanged anatomy, nor do the MUs always show a large deviation when different insets are used between pCT and CBCT. In addition, the variation of MU in the generated plans is high. Observed MU changes are reasonable given that the calculation is performed on the underlying sCT with an incorrect density distribution.

**FIGURE 5:**
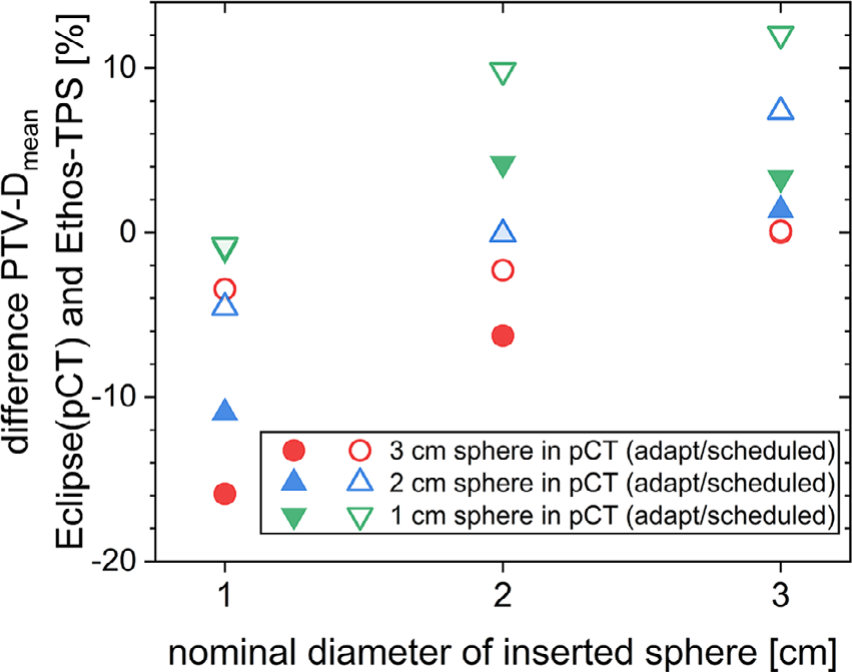
Change of PTV mean dose between the calculation in Eclipse and the calculation in the Ethos‐TPS for different combinations of inserted spheres (x‐axis) and spheres present in the initial pCT (different symbols as indicated in the legend) for treatments with both the adaptive (filled symbols) and the scheduled plan (open symbols). The data of the Ethos‐TPS are those from the treatment workstation based on the sCT. The data for Eclipse are recalculations of the adaptive plans on the pCT including the same sphere as present during the treatment session.

The mean PTV dose displayed by the Ethos‐TPS deviated from the recalculation performed on the pCT with the same inset present as during the treatment session (Figure 5). Without a change of inset, the mean doses calculated by Ethos‐TPS and Eclipse was comparable for both the adaptive and the scheduled plans for all three inset sizes—the deviation is close to 0 and the symbols are almost indistinguishable as they are on top of each other. When the insets were changed between pCT and CBCT different trends were visible for adaptive and scheduled plans. Using the scheduled plans, the dose in the Ethos‐TPS was underestimated when the inset used during the treatment session became larger than in the pCT. For the 3 cm sphere in the CBCT based on a plan with a 1 cm sphere in the pCT the difference was 12.0%. For adapted plans, the Ethos TPS typically underestimated the dose when the inset used during the treatment session became smaller than in the pCT. For the 1 cm sphere in the CBCT adapted from a plan with a 3 cm sphere in the pCT the difference was −15.9%.

### 3.2 Secondary dose calculation

**FIGURE 6:**
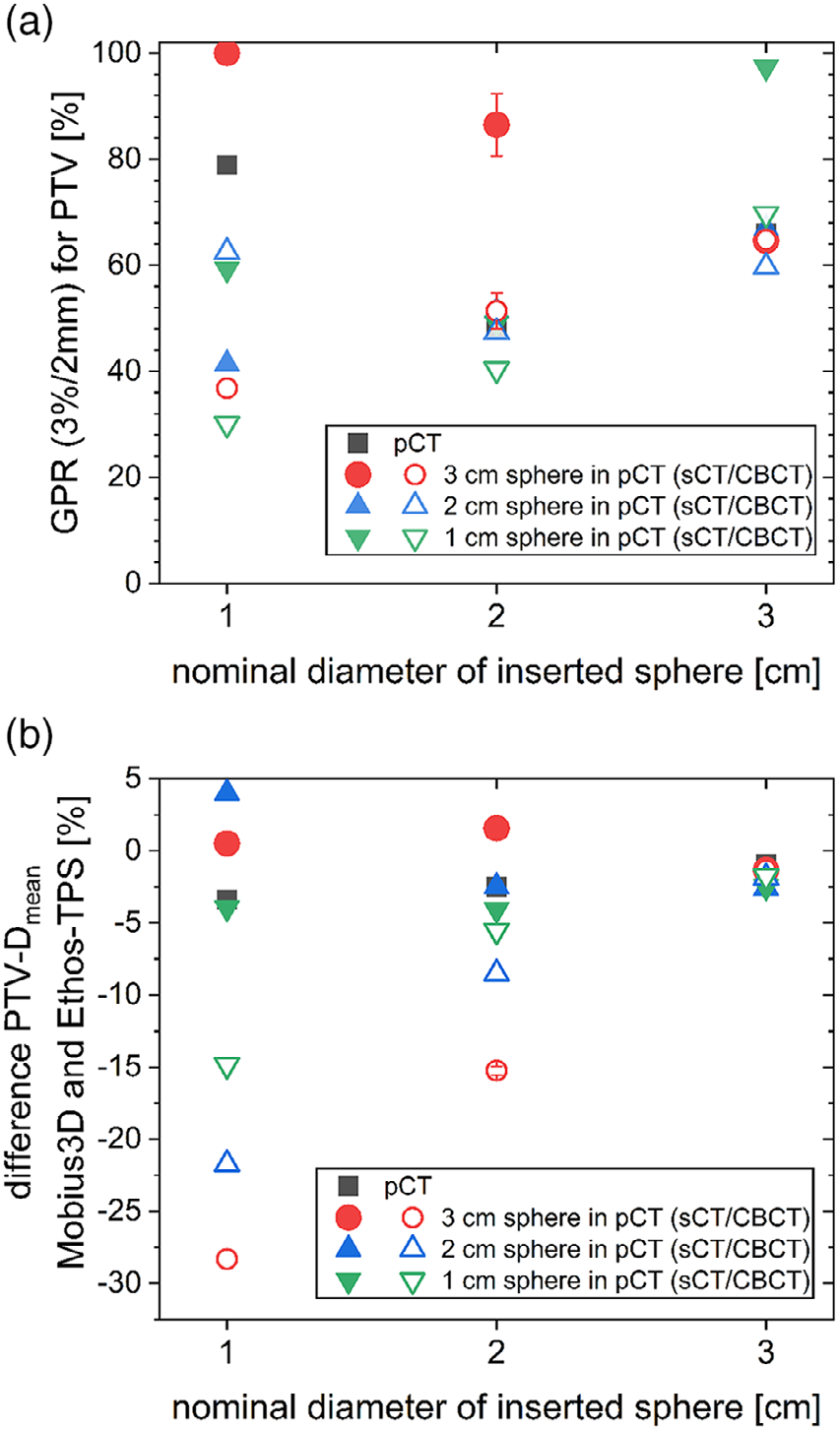
(a) GPR (3%/2 mm, absolute, no threshold, restricted to the PTV) and (b) differences in PTV mean dose between the Mobius3D recalculation and the Ethos‐TPS dose calculation for different combinations of inserted spheres (x‐axis) and spheres present in the initial pCT (different symbols as indicated in the legend) for both, calculation on the sCT (filled symbols) and the CBCT (opened symbols).

The comparison of the dose distribution and DVH parameters obtained by Mobius3D on the sCT and directly on the CBCT to those obtained with the Ethos TPS on the sCT is shown in Figure 6. The gamma passing rate (GPR) (3%/2 mm) typically decreased with decreasing diameter of the inserted sphere during the CBCT (Figure 6a). The largest variability was observed for the 1 cm sphere inserted during the CBCT. Differences between the GPR of the calculation using the sCT and the CBCT were evident for several combinations, such as a GPR (3%/2 mm) of 100% for the sCT compared to 36.8% for the CBCT calculation based on a 3 cm sphere in the pCT and a 1 cm sphere inserted at the time of treatment. The PTV mean dose calculated on the sCT in Mobius3D was within 5% of the Ethos‐TPS calculation for all combinations (Figure 6b). For the two smaller spheres inserted during treatment, the PTV mean dose obtained with Mobius3D deviated from the Ethos‐TPS dose by more than 5% in all studied combinations calculated on the CBCT. Deviations increased with the increasing size of the sphere present in the pCT and the decreasing size of the sphere inserted during the CBCT.

**FIGURE 7:**
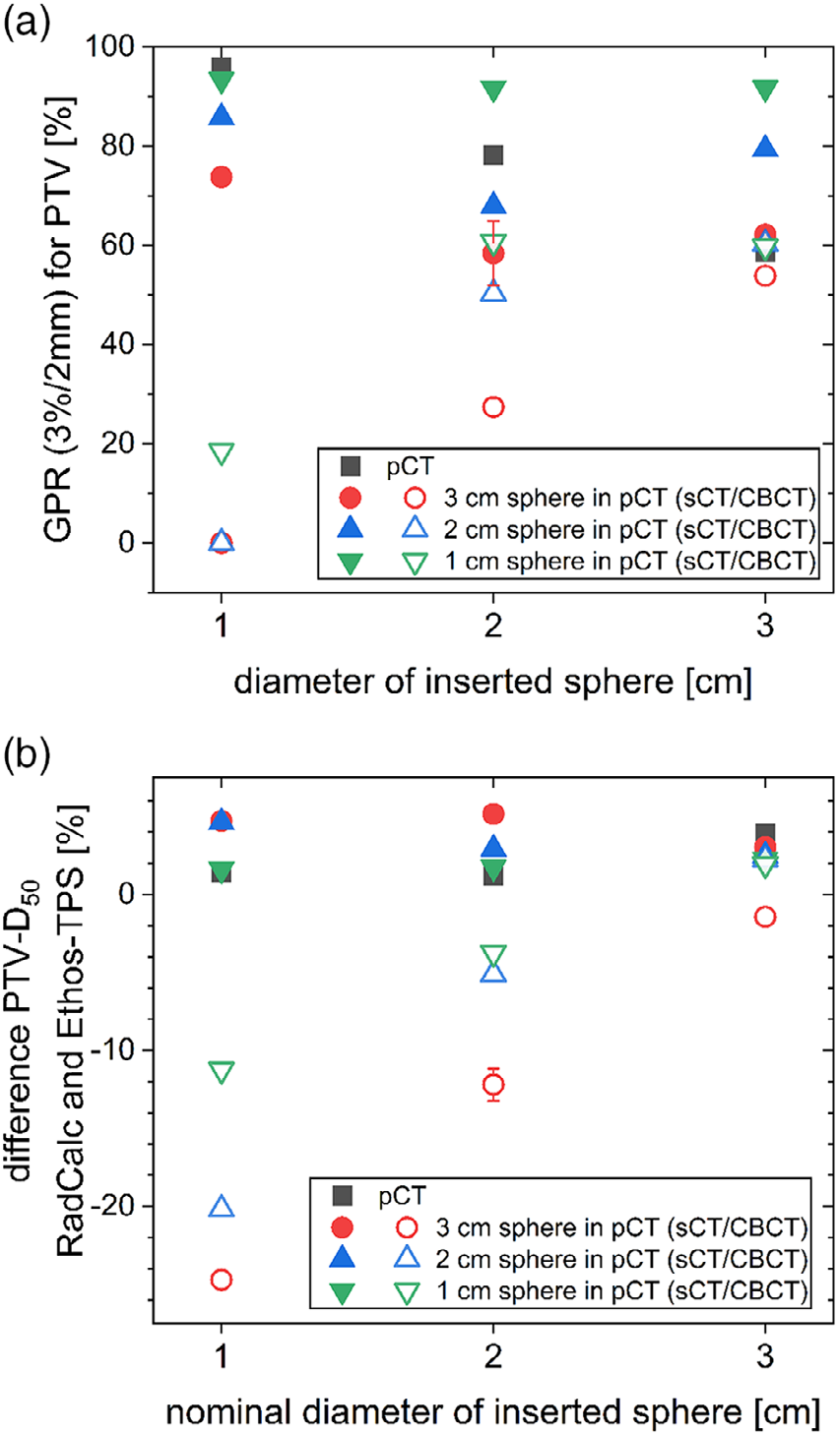
(a) GPR (3%/2 mm, absolute, no threshold, restricted to the PTV) and (b) changes in PTV median dose between the RadCalc recalculation and the Ethos‐TPS dose calculation. Different combinations of inserted spheres (x‐axis) and spheres present in the initial pCT (different symbols as indicated in the legend) were used. Calculations are either on the sCT (filled symbols) or the CBCT (opened symbol).

For RadCalc, GPR and deviations of the median dose between the secondary dose calculation and the dose distribution calculated in the Ethos‐TPS are shown in Figure 7. All calculations on the CBCT, including those with the same insets in pCT and CBCT, yielded GPR of 60.7% and lower (Figure 7a). Median doses to the PTV in the secondary calculation based on the sCT were within 3% of the dose calculated by the Ethos‐TPS. The differences were larger for calculations on the CBCT, especially for the 1 cm inset during the CBCT (Figure 7b).

**FIGURE 8:**
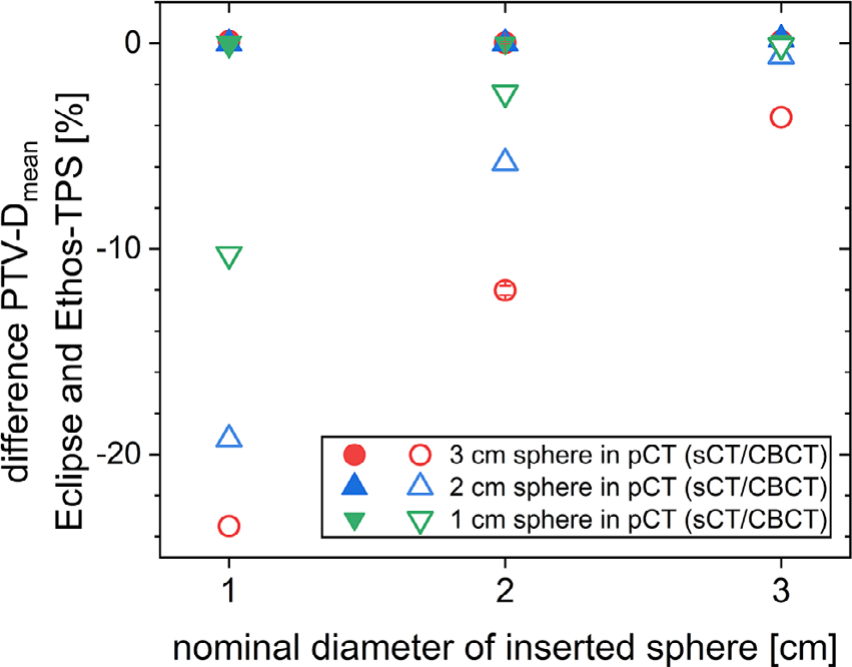
Changes in PTV mean dose between the Eclipse recalculation and the Ethos‐TPS dose calculation for different combinations of inserted spheres (x‐axis) and spheres present in the initial pCT (different symbols as indicated in the legend). Calculations are either on the sCT (filled symbols) or the CBCT (opened symbols).

Recalculations in Eclipse (Figure 8) yielded very similar results to the Ethos‐TPS when calculating on the sCT. When calculating on the CBCT, dose deviations in PTV mean dose were comparable to dose deviations for the median dose seen in RadCalc, but several percentage points off the mean doses indicated by Mobius3D.

### 3.3 EPID dosimetry

**FIGURE 9:**
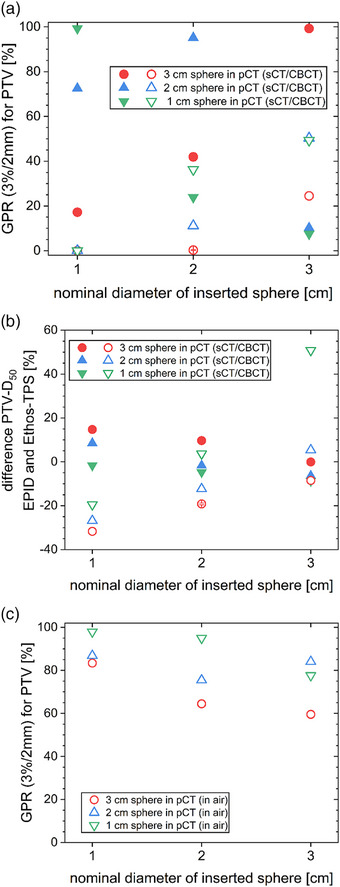
(a) GPR (3%/2 mm, absolute, no threshold, restricted to the PTV) and (b) changes in PTV median dose between the RadCalc dose distribution back‐projected from in vivo EPID images and the Ethos‐TPS dose calculation for different combinations of inserted spheres (x‐axis) and spheres present in the initial pCT (different symbols as indicated in the legend) for both calculation on the sCT (filled symbols) and the CBCT (opened symbols). (c) GPR for the EPID measurements of the respective treatment plans in air, corresponding to pre‐treatment QA without the phantom present.

GPR and median dose deviations in the PTV between dose distributions obtained by EPID dosimetry and those of the Ethos‐TPS are displayed in Figure 9. GPR (3%/2 mm) was high (>95%) for calculation on the sCT for the cases with the same inset in the pCT and CBCT, but much lower for all other combinations (Figure 9a). PTV median doses calculated by back‐projections into the sCT using EPID dosimetry were higher (up to 14.7%) than those calculated by the Ethos‐TPS for a smaller sphere inserted during CBCT than during pCT (Figure 9b). For the same combination, median doses for back‐projections into the CBCT were lower (down to −31.7%). There was a particularly large deviation for the 3 cm sphere inserted during treatment based on the plan with the pCT including the 1 cm sphere. EPID in air analysis (Figure 9c) already yielded reduced passing rates between 59.5% and 98.0%, with lower passing rates for plans created on larger targets.

## 4 DISCUSSION

The analysis of the Ethos online‐adaptive process on an anthropomorphic lung phantom, including an investigation of different QA approaches, revealed limitations of the process and especially of the QA means used for this purpose.

The automatic target contour adaptation underestimated the anatomical changes. Due to the necessity of manual contouring, the time of the adaption process is slightly longer. Surprisingly, the growth or shrinkage of the inserted sphere was not correctly represented in the sCT. A better image registration algorithm or a coupling of the registration to the user‐verified influencer and target contours would be desirable.

The following limitations apply to this study: An anthropomorphic thoracic phantom was used, which matched the typical geometry and densities, but did not include subtle anatomical details within the lung. No human CT data was used for the study, but the algorithms might work differently on actual human CT data, which could not be evaluated. It would be desirable to have the results confirmed on actual patient CTs, for example by using the emulator software available in other studies,^10^, ^11^ which unfortunately is only accessible to the vendor and selected customers. The study was performed on a static phantom, and tumor motion due to breathing would also have to be considered for clinical cases. As a further limitation, the phantom changes are not necessarily representative for the anatomical changes observed clinically. The use of the anthropomorphic phantom limited the study of anatomical changes to the available tumor insets. The introduced 1 cm diameter changes are of greater magnitude than what is observed clinically. Considerable tumor shrinkage was observed in patients with non‐small cell lung cancer, with a median gross tumor volume of 170 cm^3^ at the beginning and 100 cm^2^ at the end of radiochemotherapy.^17^ An increase in PTV can also be observed in patients with lung cancer, for example, due to pleural effusion.^18^

Given the issues observed with the sCT, it can be concluded that its accuracy must be checked routinely, ideally in such a timely manner that a reaction is possible. While the analysis revealed that the secondary dose calculation on the same sCT used for the dose calculation by the Ethos system is not able to predict errors, alternatives have been explored:

- Secondary dose calculation on the CBCT instead of the sCT,
- Recalculation on the CBCT in the TPS,
- EPID exit dosimetry (in vivo dosimetry).

Additionally, it would be helpful indicating the quality of the deformable image registration or a similarity index between CBCT and sCT, to be alarmed of unusually large discrepancies.

In this investigation, secondary dose calculation on the CBCT by Mobius3D (Figure 6a), RadCalc (Figure 7a) and in Eclipse (Figure 8) allowed to spot gross errors and also showed a reasonable size of the deviation compared to the recalculation on pCTs (Figure 5). A similar method of recalculating on CBCTs was suggested as a means of quality assurance for magnetic resonance imaging (MRI)‐only radiotherapy.^19^, ^20^ In order to be able to calculate on the CBCT for clinical cases, it should be possible to choose which CT data set is used by the QA software solutions. Alternatively, the recalculation during or at least after the treatment session in the TPS or in any third‐party software allows to verify the correct dose calculation and to initiate a reaction before treatment or at least until the next fraction. Since GPR results showed no clear trends, DVH deviations should be consulted directly to evaluate the QA. When basing any clinical decisions on recalculations on the CBCT, it should be verified that the CBCT has the required quality for dose calculation before taking any action, for example, that density values are represented correctly and that no relevant artefacts are present. When larger changes occur, it may be necessary to repeat the pCT for verification and then continue adaptive treatment from an updated baseline plan generated on the pCT to increase accuracy. It should be noted that all software solutions (planning systems as well as secondary dose calculation software) rely on generic beam models and often set the position of their dose grids automatically. Their results vary, especially for small targets and, therefore, small field sizes.

As far as EPID dosimetry is concerned, this is already widely used for in‐vivo dosimetry in radiotherapy and allows to detect a variety of anatomical changes between the time of pCT and treatment.^21^ It could also be used as a tool to verify that anatomy assumed for dose calculation of the adaptive treatments matches the planned density and to detect anatomical discrepancies between the actual patient anatomy and the anatomy assumed in the synthetic CT. Further work includes the evaluation of meaningful evaluation criteria for actual patients. If automated and evaluated in real‐time, EPID dosimetry has the potential to indicate necessary interruptions during the treatment. However, the discrepancies observed between EPID dose distributions and Ethos‐TPS dose distributions visible both in air and in phantom (Figure 9a,b) need further investigation. One possible explanation is that the provided calculation model used in RadCalc needs further consideration.

The presented data suggests that extreme care should be taken when density changes are present in the patient between pCT and CBCT at the time of treatment. This may lead to the decision to limit adaptive treatments to body regions with little changes in density. Varian recommends re‐planning rather than adaption for large anatomical changes in their handbooks, which should be taken seriously. As many scenarios are conceivable that result in density of a varying size and magnitude within a patient, we recommend additional QA procedures. In‐vivo dosimetry proved to be one option to assure that the correct dose has been delivered. Ideally, the additionally implemented QA would be automated and integrated into the standard workflow.

## 5 CONCLUSIONS

The feasibility of adaptive treatments of lung lesions on Ethos was evaluated by using an anthropomorphic phantom with different insets. Density changes were not correctly considered in the sCTs used for dose calculation and optimization.

Several QA procedures allow to reliably detect density errors in the sCT. We therefore encourage such additional QA for adaptive treatments. Re‐calculation on the CBCT prior to the treatment was demonstrated to be one feasible way. In addition, in‐vivo EPID dosimetry can be applied to detect anatomical changes between sCT and the patient anatomy during treatment.

## AUTHOR CONTRIBUTIONS

Sonja Wegener, Gary Razinskas, and Stefan Weick designed the study. Sonja Wegener, Gary Razinskas, Robert Schindhelm, and Stefan Weick obtained data and carried out the data analysis. All authors discussed the results. Sonja Wegener wrote the first draft of the manuscript. The manuscript was revised according to the input from all authors. All authors approved the final version and agree with submission.

## CONFLICT OF INTEREST STATEMENT

The authors declare no conflicts of interest.

## Data Availability

Data available on request from the corresponding author.

## References

[1] Guckenberger M , Wilbert J , Richter A , Baier K , Flentje M . Potential of adaptive radiotherapy to escalate the radiation dose in combined radiochemotherapy for locally advanced non–small cell lung cancer. Int J Radiat Oncol Biol Phys. 2011;79(3):901‐908. 20708850 10.1016/j.ijrobp.2010.04.050

[2] Møller DS , Holt MI , Alber M , et al. Adaptive radiotherapy for advanced lung cancer ensures target coverage and decreases lung dose. Radiother Oncol. 2016;121(1):32‐38. 27647459 10.1016/j.radonc.2016.08.019

[3] Kataria T , Gupta D , Bisht SS , et al. Adaptive radiotherapy in lung cancer: dosimetric benefits and clinical outcome. Br J Radiol. 2014;87(1038):20130643. 24628269 10.1259/bjr.20130643 PMC4075550

[4] Heijkoop ST , Langerak TR , Quint S , et al. Clinical implementation of an online adaptive plan‐of‐the‐day protocol for nonrigid motion management in locally advanced cervical cancer IMRT. Int J Radiat Oncol Biol Phys. 2014;90(3):673‐679. 25151538 10.1016/j.ijrobp.2014.06.046

[5] Raaymakers BW , Jürgenliemk‐Schulz IM , Bol GH , et al. First patients treated with a 1.5 T MRI‐Linac: clinical proof of concept of a high‐precision, high‐field MRI guided radiotherapy treatment. Phys Med Biol. 2017;62(23):L41‐l50. 29135471 10.1088/1361-6560/aa9517

[6] Sibolt P , Andersson LM , Calmels L , et al. Clinical implementation of artificial intelligence‐driven cone‐beam computed tomography‐guided online adaptive radiotherapy in the pelvic region. Phys Imaging Radiat Oncol. 2020;17:1‐7. 33898770 10.1016/j.phro.2020.12.004 PMC8057957

[7] Archambault Y , Boylan C , Bullock D , et al. Making on‐line adaptive radiotherapy possible using artificial intelligence and machine learning for efficient daily re‐planning. Med Phys Int 2020;8(2):77‐86

[8] Calmels L , Sibolt P , Åström LM , et al. Evaluation of an automated template‐based treatment planning system for radiotherapy of anal, rectal and prostate cancer. Tech Innov Patient Support Radiat Oncol. 2022;22:30‐36. 35464888 10.1016/j.tipsro.2022.04.001 PMC9020095

[9] Åström LM , Behrens CP , Calmels L , et al. Online adaptive radiotherapy of urinary bladder cancer with full re‐optimization to the anatomy of the day: initial experience and dosimetric benefits. Radiother Oncol. 2022;171:37‐42. 35358605 10.1016/j.radonc.2022.03.014

[10] Yoon SW , Lin H , Alonso‐Basanta M , et al. Initial evaluation of a novel cone‐beam CT‐based semi‐automated online adaptive radiotherapy system for head and neck cancer treatment—a timing and automation quality study. Cureus. 2020;12(8):e9660. 32923257 10.7759/cureus.9660 PMC7482986

[11] Mao W , Riess J , Kim J , et al. Evaluation of auto‐contouring and dose distributions for online adaptive radiation therapy of patients with locally advanced lung cancers. Pract Radiat Oncol. 2022;12(4):e329‐e338. 35219879 10.1016/j.prro.2021.12.017

[12] Liu Y , Lei Y , Wang T , et al. CBCT‐based synthetic CT generation using deep‐attention cycleGAN for pancreatic adaptive radiotherapy. Med Phys. 2020;47(6):2472‐2483. 32141618 10.1002/mp.14121 PMC7762616

[13] Stock M , Pasler M , Birkfellner W , Homolka P , Poetter R , Georg D . Image quality and stability of image‐guided radiotherapy (IGRT) devices: a comparative study. Radiother Oncol. 2009;93(1):1‐7. 19695725 10.1016/j.radonc.2009.07.012 PMC2867032

[14] Marchant TE , Moore CJ , Rowbottom CG , MacKay RI , Williams PC . Shading correction algorithm for improvement of cone‐beam CT images in radiotherapy. Phys Med Biol. 2008;53(20):5719‐5733. 18824785 10.1088/0031-9155/53/20/010

[15] Veiga C , McClelland J , Moinuddin S , et al. Toward adaptive radiotherapy for head and neck patients: feasibility study on using CT‐to‐CBCT deformable registration for “dose of the day” calculations. Med Phys. 2014;41(3):031703. 24593707 10.1118/1.4864240

[16] Kisling K , Keiper TD , Branco D , Kim GG‐Y , Moore KL , Ray X . Clinical commissioning of an adaptive radiotherapy platform: results and recommendations. J Appl Clin Med Phys. 2022;23(12):e13801. 36316805 10.1002/acm2.13801 PMC9797177

[17] Guckenberger M , Baier K , Richter A , Wilbert J , Flentje M . Evalution of surface‐based deformable image registration for adaptive radiotherapy of non‐small cell lung cancer (NSCLC). Radiat Oncol. 2009;4(1):68. 20025753 10.1186/1748-717X-4-68 PMC2804595

[18] Richter A , Weick S , Krieger T , et al. Evaluation of a software module for adaptive treatment planning and re‐irradiation. Radiat Oncol. 2017;12(1):205. 29282089 10.1186/s13014-017-0943-4 PMC5745858

[19] Palmér E , Persson E , Ambolt P , Gustafsson C , Gunnlaugsson A , Olsson LE . Cone beam CT for QA of synthetic CT in MRI only for prostate patients. J Appl Clin Med Phys. 2018;19(6):44‐52. 10.1002/acm2.12429 PMC6236859 30182461

[20] Edmund JM , Andreasen D , Mahmood F , Van Leemput K . Cone beam computed tomography guided treatment delivery and planning verification for magnetic resonance imaging only radiotherapy of the brain. Acta Oncologica. 2015;54(9):1496‐1500. 26198652 10.3109/0284186X.2015.1062546

[21] van Elmpt W , McDermott L , Nijsten S , Wendling M , Lambin P , Mijnheer B . A literature review of electronic portal imaging for radiotherapy dosimetry. Radiother Oncol. 2008;88(3):289‐309. 18706727 10.1016/j.radonc.2008.07.008

